# Electronic Patient Portal Access, Retention in Care, and Viral Suppression Among People Living With HIV in Southeastern United States: Observational Study

**DOI:** 10.2196/34712

**Published:** 2022-07-25

**Authors:** Cassandra Oliver Schember, Sarah E Scott, Cathy A Jenkins, Peter F Rebeiro, Megan Turner, Sally S Furukawa, Carmen Bofill, Zhou Yan, Gretchen P Jackson, April C Pettit

**Affiliations:** 1 Division of Epidemiology Department of Medicine Vanderbilt University Medical Center Nashville, TN United States; 2 Departments of Medicine and Pediatrics Vanderbilt University Medical Center Nashville, TN United States; 3 Department of Biostatistics Vanderbilt University Medical Center Nashville, TN United States; 4 Division of Infectious Diseases Department of Medicine Vanderbilt University Medical Center Nashville, TN United States; 5 Department of Health Information Technology Web Development Vanderbilt University Medical Center Nashville, TN United States; 6 Departments of Surgery Pediatrics and Biomedical Informatics Vanderbilt University Medical Center Nashville, TN United States

**Keywords:** HIV, viral suppression, retention in care, patient engagement, patient portal, observational study, United States, North America, eHealth, human immunodeficiency virus

## Abstract

**Background:**

Approximately 1.1 million people living with HIV live in the United States, and the incidence is highest in Southeastern United States. Electronic patient portal prevalence is increasing and can improve engagement in primary medical care. Retention in care and viral suppression—measures of engagement in HIV care—are associated with decreased HIV transmission, morbidity, and mortality.

**Objective:**

We aimed to determine if patient portal access among people living with HIV was associated with retention and viral suppression.

**Methods:**

We conducted an observational cohort study among people living with HIV in care at the Vanderbilt Comprehensive Care Clinic (Nashville, Tennessee) from 2011-2016. Individual access was defined as patient portal account registration at any point in the year prior. Retention was defined as ≥2 kept appointments or HIV lab measurements ≥3 months apart within a 12-month period. Viral suppression was defined as the last viral load in the calendar year <200 copies/mL. We calculated adjusted prevalence ratios (aPRs) and 95% CIs using modified Poisson regression with generalized estimating equations to estimate the association of portal access with retention and viral suppression.

**Results:**

We included 4237 people living with HIV contributing 16,951 person-years of follow-up (median 5, IQR 3-5 person-years). The median age was 43 (IQR 33-50) years. Of the 4237 people living with HIV, 78.1% (n=4237) were male, 40.8% (n=1727) were Black non-Hispanic, and 56.5% (n=2395) had access. Access was independently associated with retention (aPR 1.13, 95% CI 1.10-1.17) and viral suppression (aPR 1.18, 95% CI 1.14-1.22).

**Conclusions:**

In this population, patient portal access was associated with retention and viral suppression. Future prospective studies should assess the impact of increasing portal access among people living with HIV on these HIV outcomes.

## Introduction

An estimated 1.1 million people living with HIV live in the United States, and the incidence is highest in Southeastern United States [1]. The US Department of Health and Human Services announced the Ending the HIV Epidemic plan in 2019 with the goals to reduce new HIV infections by 75% by 2025 and 90% by 2030 [2,3]. To achieve these goals, the Ending the HIV Epidemic plan identified the use of rapid and effective antiretroviral therapy to achieve viral suppression as a critical component [2,3]. The HIV Care Continuum outlines the sequential steps involved in sustained viral suppression, which include (1) HIV testing and diagnosis, (2) linkage to care, (3) retention in care, (4) receipt of antiretroviral therapy, and (5) viral suppression [4]. Despite advances in HIV treatment including lower pill burden and improved tolerability, US retention and viral suppression rates remain low at approximately 50% and 56%, respectively, suggesting that barriers to HIV treatment remain [5].

Electronic patient portals are web-based tools that allow patients and their families to interact with a health care system [6,7]. Portals promote patient-centered care, where all health care decisions and quality measurements are based on an individual’s specific health needs and desired health outcomes. Electronic patient portal implementation and adoption has been rapidly increasing over the last decade [8,9]. These portals also assist health care facilities and providers in meeting the obligations of Meaningful Use within the Affordable Care Act, which requires that patients have web-based access to their health information [10]. Functionality varies across applications, but most portals allow patients to schedule appointments, access portions of their electronic health record, communicate with health care providers through secure messaging, and receive personalized health information [6,7,11].

Studies have demonstrated that electronic patient portals have increased patient engagement in care for various patient care populations and age groups [6,7,11,12]. Some studies have also assessed sociodemographic characteristics associated with patient portal use [8,13,14], such as one study that found that Black veterans living with HIV were less likely to register for and use a patient portal [15]. Few studies have assessed the impact of patient portals on HIV Care Continuum outcomes. Importantly, qualitative studies have demonstrated the acceptability of using patient portals to improve HIV care outcomes [16-18], and a study among US veterans found an association between electronic prescription refill through a patient portal and change from a detectable viral load to an undetectable viral load [19]. The objective of this study was to determine if patient portal access was independently associated with retention and viral suppression among people living with HIV engaged in care from 2011-2016 at the Vanderbilt Comprehensive Care Clinic (Nashville, Tennessee), a large HIV primary medical home in the Southeastern United States—a region disproportionately impacted by the HIV epidemic.

## Methods

### Study Population

We conducted a retrospective, observational cohort study among people living with HIV aged 18 years who had at least one HIV health care provider visit at the Vanderbilt Comprehensive Care Clinic from January 1, 2011, to December 31, 2015. The beginning of the study period was the first full year that clinic patients had access to the Vanderbilt electronic patient portal. Follow-up began on the date of the first HIV clinic visit during the study period and continued until the year prior to death or the end of the study period on December 31, 2016, allowing ≥1 year of follow-up for all people living with HIV included. We did not include data after 2016 due to a change in the Vanderbilt electronic patient portal application in 2017.

### Data Sources and Study Definitions

The Vanderbilt University Medical Center deployed a robust electronic patient portal, *My Health at Vanderbilt*, in 2005. Within 10 years of this deployment, the portal had over 290,000 registered users and was accessed over 255,000 times per month [20]. *My Health at Vanderbilt* has similar features as other electronic patient portals, including secure messaging, appointment scheduling, bill management, and access to select laboratory results and electronic health record data, and all of these features were consistently available throughout the study period [21,22]. Further description of the *My Health at Vanderbilt* patient portal can be found in descriptions of the policies and procedures [21,22].

Clinical patient data were abstracted from the electronic health record which included information collected during routine clinical care. Our exposure of interest was electronic patient portal access, defined as whether a patient was registered for a *My Health at Vanderbilt* account at any point in the year prior. To register for *My Health at Vanderbilt*, patients are required to provide their name, social security number, birth date, and a valid email address [21]. This variable was lagged by 1 year, meaning that we assessed patient portal access in the year before our outcome. This ensured that the outcomes of interest were associated with *My Health at Vanderbilt* access in the year prior in an attempt to better establish temporality between patient portal access and HIV care outcomes.

The outcomes of interest were retention and viral suppression. Retention was defined as having ≥2 maintained in-person HIV clinic appointments, HIV-1 RNA viral load measurements, or CD4+ counts which occurred ≥3 months apart within a 12-month period based on the Health and Resources Services Administration HIV/AIDS Bureau definition of retention in care [4,23,24]. Viral suppression was defined as having ≥1 HIV-1 RNA viral load measurement within a given year with the last viral load measured in the year being <200 copies/mL [4,23]. Both outcomes were measured over each 12-month period after the first clinic visit during the study period. Depending on the length of follow-up after the first visit, multiple outcomes per patient were possible. If an HIV-1 RNA viral load was missing during any 12-month interval, the patient was assumed to have a viral load of ≥200 copies/mL.

Covariates chosen based on a thorough review of the literature as well as in consultation with clinicians and epidemiologists who work directly with people living with HIV included birth sex, race/ethnicity, year of cohort entry, reported HIV transmission risk factor, insurance type, age, CD4+ cell count, and HIV-1 RNA viral load at the first clinic appointment attended. These covariates were chosen based on their connection to patient portal access and HIV care outcomes. Race/ethnicity was self-reported and categorized as White non-Hispanic, Black non-Hispanic, Hispanic, and other. Year of cohort entry was modeled continuously and defined as the year the patient entered the study. We categorized reported HIV transmission risk factors as male-male sexual contact (men who have sex with men; MSM), heterosexual contact, injection drug use (IDU), or other/unknown. If a patient had more than one type of transmission risk, IDU took precedence over MSM, which took precedence over heterosexual contact, in order of the risk of HIV transmission [25]. Insurance type was categorized as public (Medicare/Medicaid), private, or Ryan White. If an individual had more than one insurance type in a given year, Ryan White took precedence over public insurance, which took precedence over private insurance. Baseline CD4+ count was defined as the laboratory measurement closest to the first maintained appointment date (from 180 days prior to 30 days after); it was square-root transformed, modeled as a continuous covariate in the regression model, and displayed in our tables using the clinically salient CD4+ values of 100, 200, 350, and 500 cells/µL. Baseline HIV-1 RNA viral load was similarly defined as the laboratory measurement closest to the first maintained appointment date (from 180 days prior to 7 days after); it was log_10_-transformed and modeled continuously. Insurance status was time-updated during each 12-month period after enrollment. The remaining covariates were measured only at baseline.

### Statistical Analysis

We reported demographic characteristics stratified by the existence of a patient portal account during follow-up, as we wanted to compare those who never accessed the patient portal to those who did. We reported categorical variables by frequency and proportion and used Pearson chi-squared test for comparisons. Continuous variables were reported as median and IQR, and Wilcoxon rank sum tests were used for comparisons [26,27]. Multiple imputation with 10 replications was used to account for missing CD4+ cell counts and HIV-1 RNA viral loads at baseline [28]. If missing, the reported HIV transmission risk factor was assumed to be other/unknown, and insurance type was handled by carrying forward the last observation. No patient was missing insurance type at baseline.

We estimated adjusted prevalence ratios (aPRs) and 95% CIs for retention and viral suppression using a modified Poisson regression [29]. Generalized estimating equations using an independence correlation structure accounted for multiple outcomes per individual [30,31]. A clustered sandwich estimator was used to estimate SEs [32-34]. In a sensitivity analysis, we excluded individuals with missing data to assess if a complete case analysis biased our results. All tests were 2-tailed and considered statistically signiﬁcant if *P*<.05. All analyses were conducted using R statistical software (version 3.4; R Foundation for Statistical Computing).

### Ethics Approval

Analyses were approved by the Vanderbilt University Institutional Review Board (approval number 170089) and conducted in accordance with the ethical standards set by the Declaration of Helsinki.

## Results

### Demographic Characteristics

The study population included 4237 people living with HIV followed for a total of 16,951 person-years. Of the 16,951 person-years, 74.8% (n=12,679) were categorized as retained in care and 71.4% (n=12,103) as virally suppressed. Median follow-up time per patient was 5 (IQR 3-5) person-years. The median age was 43 (IQR 33-50) years. Of the 4237 people living with HIV, 78.1% (n=3311) were male, 40.8% (n=1727) were Black non-Hispanic, and 41.2% (n=1747) reported MSM as an HIV transmission risk factor. The median baseline CD4+ count was 478 (IQR 288-692) cells/µL and median baseline HIV-1 RNA viral load was 100 (IQR 50-25,119) copies/mL (Table 1). Of the 4237 people living with HIV, reported HIV transmission risk factor, baseline CD4+ count, and HIV-1 RNA viral load were missing for 30.8% (n=1305), 34.2% (n=1449), and 44.8% (n=1898) of the participants, respectively. Insurance type varied over time; of the 16,951 person-years, 21% (n=3560) had private insurance, 40.1% (n=6797) had Ryan White, 27.7% (n=4695) had public insurance, and 11.2% (n=1899) were missing for which the last observation was carried forward.

Of the 4237 people living with HIV included, 56.5% (n=2395) had patient portal access at any point during follow-up. People living with HIV who had a *My Health at Vanderbilt* account were younger, with a median age of 42 (IQR 31-49) years, than those without an account, who had a median age of 44 (IQR 34-51) years. This difference was statistically significant, but a difference of 2 years is arguably not a clinically significant difference. A higher percentage (85.6%, 2050/2395) of those with an account were male, whereas only 68.5% (1261/1842) of those without an account were male. Fewer people living with HIV with patient portal access (30.2%, 724/2395) were Black non-Hispanic than people living with HIV without access (54.5%, 1003/1842). More people living with HIV with access (52.2%, 1250/2395) reported their HIV transmission risk factor as MSM than those without access (27%, 497/1842). Those with access also had a higher median baseline CD4+ count of 500 (IQR 309-702) cells/µL than those without access, who had a median baseline count of 444 (IQR 258-676) cells/µL. The baseline HIV-1 RNA viral load was similar between these 2 groups (Table 1).

**Table 1:** Baseline demographic characteristics of the study population stratified by patient portal account status.

Characteristic			No account during follow-up (n=1842)		Account existed during follow-up (n=2395)		All participants (N=4237)		*P* value^a^	
Baseline age (years), median (IQR)			44 (34-51)		42 (31-49)		43 (33-50)		<.001	
**Sex, n (%)**										<.001
	Male	1261 (68.5)		2050 (85.6)		3311 (78.1)				
	Female	581 (31.5)		345 (14.4)		926 (21.9)				
**Race/ethnicity, n (%)**										<.001
	Black non-Hispanic	1003 (54.5)		724 (30.2)		1727 (40.8)				
	Hispanic	138 (7.5)		102 (4.3)		240 (5.7)				
	White non-Hispanic	407 (22.1)		1134 (47.3)		1541 (36.4)				
	Other/unknown	294 (16)		435 (18.2)		729 (17.2)				
**HIV risk factor, n (%)**										<.001
	MSM^b^	497 (27)		1250 (52.2)		1747 (41.2)				
	Heterosexual	622 (33.8)		332 (13.9)		954 (22.5)				
	IDU^c^	80 (4.3)		35 (1.5)		115 (2.7)				
	Other/unknown	66 (3.6)		50 (2.1)		116 (2.7)				
	Missing data	577 (31.3)		728 (30.4)		1305 (30.8)				
Baseline CD4+ count (cells/µL), median (IQR)			444 (258-676)		500 (309-702)		478 (288-692)		<.001	
Baseline HIV-1 RNA viral load (copies/mL), median (IQR)			158.5 (50.1-19,952.6)		63.1 (50.1-25,118.9)		100.0 (50.1-25,118.9)		.30	
**Year of cohort entry, n (%)**										.007
	2011	1126 (61.1)		1452 (60.6)		2578 (60.8)				
	2012	155 (8.4)		234 (9.8)		389 (9.2)				
	2013	175 (9.5)		211 (8.8)		386 (9.1)				
	2014	198 (10.7)		236 (9.9)		434 (10.2)				
	2015	146 (7.9)		235 (9.8)		381 (9)				
	2016	42 (2.3)		27 (1.1)		69 (1.6)				

^a^Wilcoxon rank sum test was used for continuous variables and Pearson chi-square test was used for categorical variables to compare those with an account to those without an account.

^b^MSM: men who have sex with men.

^c^IDU: injection drug use.

### Retention in Care Outcome

In the multiple imputed, adjusted, and modified Poisson regression analysis, patient portal access was independently associated with better retention (aPR 1.13, 95% CI 1.10-1.17; Table 2). Other factors independently associated with better retention in this model included increased age at first visit (aPR 1.09, 95% CI 1.04-1.13) and MSM (aPR 1.13, 95% CI 1.03-1.23) and heterosexual contact (aPR 1.15, 95% CI 1.05-1.26) as reported HIV transmission risk factors compared to IDU (Table 2). A factor independently associated with worse retention was other/unknown race/ethnicity as compared to White non-Hispanic (aPR 0.93, 95% CI 0.90-0.97; Table 2).

**Table 2:** Adjusted prevalence ratios for the association of patient portal account existence and HIV outcomes of retention in care and viral suppression. All models adjusted for variables included in the table as well as the year of cohort entry.

Characteristic		Retention in care model, aPR^a^ (95% CI)		Viral suppression model, aPR (95% CI)	
**Account status** **(variable lagged by 1 year)**					
	No account		REF^b^		REF
	Account exists		1.13 (1.10-1.17)*		1.18 (1.14-1.22)*
Baseline age (per 10 years)			1.09 (1.04-1.13)*		1.09 (1.04-1.13)*
**Sex**					
	Male		REF		REF
	Female		1.04 (1.00-1.08)		0.99 (0.95-1.04)
**Race/ethnicity**					
	Black non-Hispanic		0.99 (0.95-1.02)		0.95 (0.92-0.99)*
	Hispanic		1.04 (0.98-1.11)		1.03 (0.96-1.10)
	White non-Hispanic		REF		REF
	Other/unknown		0.93 (0.90-0.97)*		0.94 (0.90-0.97)*
**HIV risk factor**					
	MSM^c^		1.13 (1.03-1.23)*		1.11 (1.00-1.23)
	Heterosexual		1.15 (1.05-1.26)*		1.15 (1.03-1.27)*
	IDU^d^		REF		REF
	Other/unknown		0.96 (0.88-1.05)		0.95 (0.86-1.06)
**Insurance**					
	Private		REF		REF
	Public		1.03 (0.99-1.07)		0.97 (0.94-1.01)
	Ryan White		0.99 (0.95-1.02)		0.94 (0.90-0.98)*
**Baseline CD4+ count (square-root transformed; cells/µL)**					
	100		0.99 (0.91-1.07)		0.99 (0.91-1.07)
	200		0.99 (0.97-1.02)		0.99 (0.97-1.02)
	350		REF		REF
	500		1.01 (0.99-1.02)		1.00 (0.99-1.02)
Baseline HIV-1 RNA viral load (log_10_-transformed; copies/mL)			1.00 (0.98-1.01)		0.94 (0.92-0.96)*

^a^aPR: adjusted prevalence ratio.

^b^REF: reference.

^c^MSM: men who have sex with men.

^d^IDU: injection drug use.

*P<.05.

### Viral Suppression Outcome

In the multiple imputed, adjusted, and modified Poisson regression analysis, patient portal access was independently associated with improved viral suppression (aPR 1.18, 95% CI 1.14-1.22; Table 2). Other factors independently associated with better viral suppression included increased age at first visit (aPR 1.09. 95% CI 1.04-1.13) and heterosexual contact as a reported HIV transmission risk factor as compared to IDU (aPR 1.15, 95% CI 1.03-1.27). Factors independently associated with worse viral suppression included Black non-Hispanic (aPR 0.95, 95% CI 0.92-0.99) and other/unknown (aPR 0.94, 95% CI 0.90-0.97) race/ethnicity as compared to White non-Hispanic race/ethnicity; Ryan White coverage as compared to private insurance (aPR 0.94, 95% CI 0.90-0.98); and higher HIV-1 RNA viral load at first clinic visit (aPR 0.94, 95% CI 0.92-0.96; Table 2).

### Sensitivity Analysis

We conducted a sensitivity analysis in which patients with missing data were excluded. This led to a complete case population of 1643 patients (38.8% of total cohort, N=4237) contributing 5589 person-years (33% of total person-years, N=16,951). The results were similar, but less precise, when the 2 full models from the primary analysis were used for retention and viral suppression (Table 3). Patient portal access remained associated with increased likelihood of retention (aPR 1.13, 95% CI 1.07-1.19) and viral suppression (aPR 1.16, 95% CI 1.10-1.23; Table 3).

**Table 3:** Adjusted prevalence ratios for the association of patient portal account existence and the HIV outcomes of retention in care and viral suppression—complete case analysis. All models adjusted for variables included in the table as well as the year of cohort entry.

Characteristic		Retention in care model, aPR^a^ (95% CI)	Viral suppression model, aPR (95% CI)
**Account status (variable lagged by 1 year)**			
	No account	REF^b^	REF
	Account exists	1.13 (1.07-1.19)*	1.16 (1.10-1.23)*
Baseline age (per 10 years)		1.08 (1.06-1.10)*	1.08 (1.06-1.11)*
**Sex**			
	Male	REF	REF
	Female	1.05 (0.98-1.13)	1.01 (0.93-1.09)
**Race/ethnicity**			
	Black non-Hispanic	0.96 (0.97-1.01)	0.92 (0.87-0.98)*
	Hispanic	1.00 (0.91-1.10)	0.97 (0.87-1.08)
	White non-Hispanic	REF	REF
	Other/unknown	0.95 (0.88-1.01)	0.96 (0.90-1.03)
**HIV risk factor**			
	MSM^c^	1.06 (0.93-1.21)	1.05 (0.92-1.20)
	Heterosexual	1.12 (0.98-1.28)	1.12 (0.97-1.29)
	IDU^d^	REF	REF
	Other/unknown	1.01 (0.85-1.20)	1.01 (0.85-1.21)
**Insurance**			
	Private	REF	REF
	Public	0.97 (0.91-1.04)	0.94 (0.88-1.01)
	Ryan White	1.00 (0.95-1.05)	0.96 (0.90-1.01)
**Baseline CD4+ count (square-root transformed; cells/µL)**			
	100	1.00 (0.98-1.03)	0.99 (0.98-1.05)
	200	1.00 (0.99-1.02)	0.99 (0.99-1.02)
	350	REF	REF
	500	1.00 (0.99-1.01)	1.01 (0.99-1.02)
Baseline HIV-1 RNA viral load (log_10_-transformed; copies/mL)		1.00 (0.98-1.02)	0.96 (0.94-0.98)*

^a^aPR: adjusted prevalence ratio.

^b^REF: reference.

^c^MSM: men who have sex with men.

^d^IDU: injection drug use.

*P<.05.

## Discussion

### Principal Findings

Electronic patient portal access via Vanderbilt’s *My Health at Vanderbilt* system was significantly associated with subsequent retention and viral suppression among people living with HIV in care at the Vanderbilt Comprehensive Care Clinic. This finding is consistent with previous findings from a Kaiser Permanente study that found patient portals increased patient membership retention for both people living with HIV and people not living with HIV [35]. There have been other studies of people living with HIV that found patient portals improve retention and viral suppression, but these were in less diverse or much smaller patient populations [16,19]. A small (n=22) prospective quality improvement project aimed to increase enrollment in a patient portal among women living with HIV to improve their retention in HIV care, given their increased risk of disengagement [16]. The authors found a significant association between enrollment in the patient portal and the number of scheduled visits but did not find a significant association with missed visits or viral suppression [16]. Another retrospective study among a population of 3374 veterans living with HIV found a significant association between messaging from a personal health record and viral suppression, but the authors did not assess retention or how patient portal access affected viral suppression [19]. The strengths of our study include having a large, demographically diverse cohort of people living with HIV living in Southeastern United States, a region of the country disproportionately affected by the HIV epidemic.

In our cohort, compared to patients without patient portal access, those with access were more likely to be younger, male, White non-Hispanic, and report MSM as their HIV transmission risk factor. They also had a higher CD4+ count at their first clinic visit compared to patients without patient portal access. Our results are consistent with previous studies in populations including people living with HIV and people without HIV, which showed that a higher proportion of those with access to patient portals tend to be younger and White, although the age difference in our study was only 2 years [8,11,13,14]. These differences could be due to increased technological literacy in using computers and smartphones [8,14]. Sex differences in patient portal access in other studies have varied, with some showing that women access patient portals more, and others showing that men preferred using patient portals than speaking in person with their health care providers [8,13]. In our cohort, men were more likely to have patient portal access. Our cohort had a higher proportion of men, but if there were no sex differences, we would expect the same proportion among those with and without access.

In addition to patient portal access, increasing age and reported HIV risk factor were independently associated with retention and viral suppression. People living with HIV in an older age group compared to those in a younger age group and people living with HIV who reported heterosexual activity or MSM compared to IDU as an HIV transmission risk factor were more likely to achieve retention and viral suppression. These findings are consistent with a systematic review of retention studies [36].

Factors that were independently associated with worse retention and viral suppression included race/ethnicity, insurance type, and HIV-1 RNA viral load at the first Vanderbilt Comprehensive Care Clinic visit. People living with HIV who are Black non-Hispanic (compared to those who are White non-Hispanic), have Ryan White coverage (compared to private insurance), or had a higher HIV-1 RNA viral load at their first clinic visit had worse retention and viral suppression. These findings are consistent with previous cohort studies assessing viral suppression trends over time, in which Black non-Hispanic race/ethnicity was associated with worse viral suppression and having Ryan White insurance was associated with worse HIV outcomes [37,38]. However, worse outcomes for people receiving care via the Ryan White HIV/AIDS Program is likely because it is a proxy for lower socioeconomic status. Our findings show that patient portal access follows similar trends to disparities in HIV care outcomes by age, race, HIV transmission risk factor, and insurance status, as groups with poor patient portal access also have poor HIV outcomes.

In the setting of the current COVID-19 pandemic, engaging care through electronic means such as patient portals and telehealth have increased [39,40]. This pandemic may have lasting effects on how individuals access and engage care, showing the importance of better understanding the effects of patient portal access on HIV care outcomes.

Our study is subject to several limitations. First, we had data on patient portal access but not on the frequency of or reasons for electronic patient portal use. It is possible to have patient portal access but never use the portal. However, regardless of use, patient portal access was associated with improved retention and viral suppression, demonstrating that providing access to patient portals is likely to improve HIV outcomes. Similarly, studies have stressed the importance of electronic health literacy in patient portal effectiveness and care outcomes. In our study, a patient may have had access to the electronic patient portal and used it but also had difficulty understanding the platform or information due to technological or health literacy barriers [41,42]. Both scenarios would have biased our results toward a null hypothesis; therefore, it is possible that the true relationship between patient portal access and retention and viral suppression may be stronger than what we described. Second, some people living with HIV in our cohort may have silently transferred to other clinics, which led them to be misclassified in our study as not retained in care. This may have led to an overestimation of those not retained in care, which could have biased our results in either direction depending on the population misclassified. Third, the reported HIV transmission risk factor, baseline CD4+ count, and baseline HIV-1 RNA viral load were missing for 31% to 45% of participants. The missing data for this risk factor and baseline measures of clinical variables were accounted for with multiple imputation. The results of the sensitivity analysis including only patients with complete records had similar results, suggesting that data were missing completely at random and therefore not a likely source of bias. Additionally, this was a single-site study and may not be generalizable to other settings, as electronic patient portal access may differ elsewhere. Lastly, these data are from 2011-2016. We were unable to provide more recent data because after 2016, Vanderbilt’s patient portal changed. However, we were still able to establish a connection between an early patient portal and favorable HIV outcomes.

We examined the association of an under-studied exposure with HIV care outcomes and found that electronic patient poral access was independently associated with retention and viral suppression in our cohort of people living with HIV. Studies have demonstrated that electronic patient portals offer a unique opportunity to improve outcomes that are a part of the HIV Care Continuum, such as retention and viral suppression [17,18]. Our study supports prior findings and fills a gap in previous literature by examining this association in a large cohort of people living with HIV in an area disproportionately affected by HIV with a median longitudinal follow-up of 5 years.

### Conclusions

Retention and viral suppression are necessary for reducing HIV transmission and mortality, as well as increasing the quality of life for people living with HIV. We found that electronic patient portal access was associated with improved retention and viral suppression. This suggests that increased access to electronic patient portals among people living with HIV may be an effective method to promote better HIV Care Continuum outcomes. Large prospective studies assessing the impact of patient portal access on retention and viral suppression are needed to confirm these findings.

## Abbreviations

aPR: adjusted prevalence ratio
IDU: injection drug use
MSM: men who have sex with men
